# Surgical Skill Decay as a Result of the COVID-19 Pandemic

**DOI:** 10.3390/life14081020

**Published:** 2024-08-16

**Authors:** Natalia Olszewska, Tomasz Guzel, Thomas Carus, Maciej Słodkowski

**Affiliations:** 1 Department of General, Gastroenterological and Oncological Surgery, Central Clinical Hospital of Medical University of Warsaw, 02-091 Warsaw, Poland; natalia.olszewska@wum.edu.pl (N.O.); maciej.slodkowski@wum.edu.pl (M.S.); 2 German-Polish Association of MiniInvasive Surgery, 02-091 Warsaw, Poland; t.carus@kliniken-lkd.de; 3 Department of General and Visceral Surgery, Bassum Clinic, 27211 Bassum, Germany

**Keywords:** surgical skill decay, COVID-19 pandemic, resident training, surgical education, laparoscopic cholecystectomy

## Abstract

Background: This study aimed to objectively evaluate the impact of the gap in surgical practice caused by COVID-19 on surgical skill decay. Methods: This retrospective cohort study enrolled 148 cases of adult patients who were qualified for elective or urgent laparoscopic cholecystectomy. This study compared the period of nine months before the pandemic outbreak and nine months after the end of the pandemic. We analyzed the duration of surgery, the number of intraoperative adverse events (IAEs), postoperative complications (PCs), and differences between the surgeries performed by residents and those performed by specialists. Results: The number of IAEs did not differ significantly between groups (after COVID-19 (AC) and before COVID-19 (BC)). A difficult gallbladder (DGB) was associated with an increased risk of IAEs during surgery in both groups (BC:OR = 2.94, *p* = 0.049; AC:OR = 2.81, *p* = 0.35). The multivariate analyses showed no significant differences in IAEs or PCs when the residents performed surgeries compared to specialists. The average duration of surgery was significantly longer in the post-pandemic group (BC–102.4 min vs. AC–119.9 min, *p* = 0.024). Conclusions: Measurable determinants of surgical skills are the duration of surgery and the number of intraoperative adverse events. By defining this indicators, our study objectively shows that the reduction in the volume of surgeries during COVID-19 resulted in a phenomenon known as surgical skill decay.

## 1. Introduction

The first case of SARS-CoV-2 infection was confirmed in December 2019 in the Chinese city of Wuhan [1,2]. Within weeks, the COVID-19 pandemic (COVID-19) spread to each continent, completely changing the world’s functioning as we had known it until then. In Poland, patient zero was diagnosed on 4 March 2020. Up until now, more than 704 million people have been infected worldwide, of whom nearly 7 million died [3,4,5]. 

COVID-19 has significantly affected the organization of health care systems worldwide. Due to the high risk of SARS-CoV-2 infection among staff and patients during hospitalization, necessary modifications were made in the functioning of surgical departments and operating theaters [6,7]. This entirely changed the profile of patients treated by surgeons and drastically reduced the time spent in the operating theater. The average number of laparoscopic cholecystectomies performed in our department during COVID-19 period was reduced by more than 50% compared to the pre- and post-pandemic periods. The reduction in elective procedures led to a significant decrease in the number of performed surgeries, which had a huge impact on residents’ surgical training [4,6,8,9,10]. Furthermore, the reassignment of surgical specialty residents to work in isolation units, treating coronavirus-infected patients, also resulted in fewer opportunities to develop surgical skills [6,7,11]. Moreover, the enormous mental burden of being responsible for patients’ lives, coping with feelings of burnout, post-traumatic stress disorder, and depression left a significant mark on the pursuit of careers, for both young and experienced doctors [6,8,9,10,11].

Through the lack of sufficient surgical practice, a phenomenon described in the literature as surgical skill decay may occur [12,13]. Skill decay is defined as the loss of acquired technical skills or knowledge caused by an extended period of nonuse. Physical, speed-based, and pattern-based tasks are more likely to result in skill loss than accuracy-based and out-of-the-box tasks that require the use of cognitive intelligence [12,14]. Additionally, the severity of the loss of acquired skills is directly correlated to the length of time during which they have been unused. Hence, greater knowledge and better-trained skills prior to the onset of a break are associated with a less intense skill decay effect [12]. 

The medical literature mainly describes this phenomenon among residents who take a gap year dedicated to research work, surgeons forced to take an extended career break due to personal reasons, illness, or pregnancy, and specialists who undertake military duty, performing exclusively combat-field surgeries [13,15,16,17]. However, the available publications show that the loss of acquired surgical skills due to the COVID-19 pandemic has not been objectively studied and evaluated, despite several surveys which subjectively assessed the influence of the coronavirus pandemic on the occurrence and severity of surgical skill decay [4,8,9,10,11,18,19,20,21,22,23,24].

The aim of this study is to objectively evaluate the impact of the break in normal surgical practice caused by COVID-19 on surgical skill decay, analyzing laparoscopic cholecystectomies as an example.

## 2. Materials and Methods

We conducted a retrospective cohort study. Consecutive sampling was applied to the patients enrolled in this study. The patients who were initially qualified for a laparoscopic cholecystectomy and whose surgery began laparoscopically were recruited. We selected 148 patients who had been admitted for either urgent or elective laparoscopic cholecystectomies at the Department of General, Gastroenterological and Oncological Surgery of the Medical University of Warsaw. We compared the period of 9 months before the pandemic outbreak (BC–76 patients) and 9 months after the end of the pandemic (AC–72 patients). The before COVID-19 (BC) group consisted of patients admitted from 20 June 2019 to 20 March 2020 (9 months), while the after COVID-19 (AC) group included patients admitted from 13 May 2022 to 13 February 2023 (9 months). The sample size reflects the number of operations performed in the same period before and after the COVID-19 gap. Thus, the determination of the sample size in advance was not possible; nevertheless, the number of patients in the groups before COVID (BC) and after COVID (AC) was comparable (BC = 76 vs. AC = 72). Clinical data and laboratory parameters were collected by analyzing the patients’ medical histories available in the hospital network systems and paper medical records. In Poland, the outbreak was declared on 20 March 2020 and lasted until 13 May 2022. (i.e., 27 months). During the entire COVID-19 pandemic period, following the directives of the Ministry of Health [25], no elective surgeries were performed, excluding surgeries for oncological indications. This aligned with practices in other university hospitals worldwide [4,8,10,11,22].

We specifically investigated selected outcomes, namely, the necessity of conversion, the duration of surgery, the hospitalization time, and the occurrence of intraoperative adverse events (IAEs), with these events being defined as one of the following:

- Injury of the gallbladder wall;
- Evacuation of gallstones into the peritoneum;
- Intraoperative bleeding with no need for conversion to laparotomy.

We also considered the occurrence of postoperative complications (PC) such as the following:

- Postoperative fluid collection;
- Biliary leakage;
- Postoperative bleeding;
- Wound infection;
- Necessity of readmission to the hospital;
- Necessity of reoperation.

Moreover, to simplify and systematize several statistical analyses, after the collection and initial clustering of data, we introduced and defined the term “difficult gallbladder” (DGB) if any of the following was noted in the operation reports:

- Enlarged gallbladder;
- Hard gallbladder wall;
- Thick gallbladder wall;
- Inflammation seen during surgery;
- Intraperitoneal adhesions.

It is worth noting that the cholecystectomies, both before and after COVID-19, were performed by exactly the same teams of surgeons, whether specialists or residents. Residents are surgeons undergoing their surgical training, which lasts 6 years in Poland. Specialists are experienced surgeons who have performed more than 500 surgeries (including laparoscopic cholecystectomies).

The group characteristics are summarized in Table 1, using percentages for the categorical variables and the median with the interquartile range (IQR) or the mean with the standard deviation (SD) for the quantitative variables. Fisher’s exact test and the Mann–Whitney test were used to compare groups for qualitative and quantitative variables, respectively. Logistic regression was used to estimate the risk of adverse events according to the operative conditions or demographic data. To estimate the influence of the duration of surgery on the intraoperative adverse events independent of other concomitant variables, a series of two-factor logistic regressions were performed. A statistical analysis was performed using SAS 9.4 (SAS Institute, Cary, NC, USA; 2022). *p*-values of <0.05 were considered statistically significant.

## 3. Results

This research shows that, demographically, the groups of patients studied before and after the pandemic were not statistically different. Men were the minority in both groups, and the median age, body mass, and BMI of the patients were comparable. The number of acute cholecystectomies was significantly higher BC (35.5%) than AC (20.8%), with *p* = 0.047 (Table 1). Twelve patients in group BC (15.8%) and eight patients in group AC (11.1%) received a postoperative antibiotic treatment, but the difference is not statistically significant, with *p* = 0.249 (not included in the tables).

The necessity of conversion in group BC was 18.4%, while, in group AC, it was 8.3%, with *p* = 0.07. A comparable number of postoperative complications were observed in the BC and AC groups (13.2% and 9.7%, respectively), and the difference is not significant, with *p* = 0.51. One patient in the AC group required reoperation, due to iatrogenic perforation of the small intestine. It is worth noting that patients in neither group experienced serious postoperative complications such as biliary injury, postoperative hemorrhage, or hepatic vascular damage. The average hospitalization time was longer in the BC group than in the AC group by 0.6 days (BC–6.2 vs. AC–5.6), with *p* = 0.011 (Table 2).

A significantly higher percentage of difficult gallbladders was found BC compared to AC (69.7% vs. 47.2%, respectively), with *p* = 0.005 (Table 1). DGB was associated with a more than double risk of intraoperative adverse events (BC: odds ratio (OR) = 2.94, *p* = 0.049; AC: OR = 2.81, *p* = 0.035). Moreover, a significant independent factor for the occurrence of IAEs in both groups was a difficult gallbladder (*p* < 0.05). The risk of conversion for the open procedure was higher among the patients with an acute gallbladder (BC: OR = 10.5, *p* = 0.001; AC: OR–4.5, *p* = 0.09) (Table 3).

The number of IAEs did not differ significantly between the two groups in the univariate analysis. The multivariate analysis indicated that, after excluding the influence of the DGB, the risk of IAEs increased (adjusted odds ratio (aOR) = 1.38 vs. aOR = 1.80, *p* = 0.100) in the AC group. In the multivariate analyses, there were no significant differences in the IAEs or PCs between the cases when the residents performed the surgeries compared to the specialists (Table 4). 

In group BC, specialists served as the lead operators in a higher proportion of surgeries compared to group AC (BC 65.8% vs. AC 58.3%), but the difference is not statistically significant, with *p* = 0.35 (Table 1). In both groups, the duration of surgery was shorter when performed by specialists, as opposed to the procedures led by residents. Furthermore, a difficult gallbladder and acute surgery were risk factors for a longer duration of surgery in both groups and reached statistical significance in the BC group. (Table 5).

**Figure 1:**
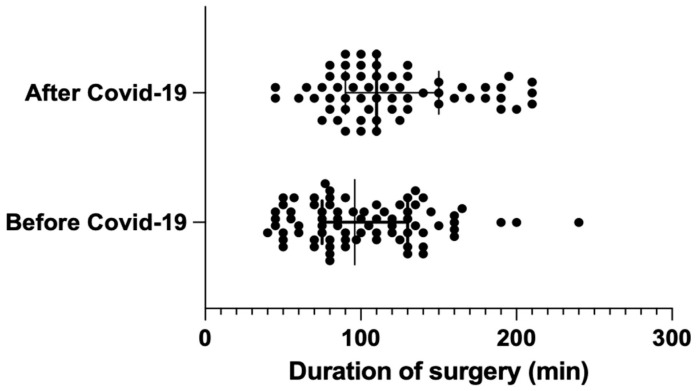
Distribution of surgery duration before and after COVID-19 (median with interquartile range).

The average duration of surgery was significantly longer (by 17.5 min) in the post-pandemic group, with group AC measuring 119.9 min vs. group BC measuring 102.4 min (BC: median = 96 min [75; 130] vs. AC: median = 110 min [95; 150]); the result was statistically significant, with *p* = 0.024. (Figure 1).

## 4. Discussion

Upon reviewing the available literature, we did not identify any study that objectively assessed the impact of COVID-19 on surgical performance skills. Most publications primarily focused on analyzing survey questionnaires where doctors subjectively evaluated their experiences during the peri-pandemic period [8,9,10,11,18,19,20,21,22,23,24]. Their specialty training suffered significantly because of the COVID-19 pandemic. Moreover, nearly 70% of the survey’s respondents admitted to feeling an impairment in their surgical skills and confidence [9,10,11,18,19,20,21,22,23,24]. The unique circumstances precipitated by the COVID-19 pandemic have provided a rare opportunity to assess the impact of prolonged breaks in surgical practice on skill retention. Our findings hold universal relevance, as analogous disruptions were experienced across health care systems worldwide [18,19,20,21,22,23,24]. By shedding light on the concept of surgical skill decay, we hope to start discussions and initiatives aimed at mitigating its effects, thereby enhancing patient outcomes and improving surgeon confidence.

According to multiple studies, a measurable determinant of surgical efficiency includes the time taken to perform surgeries and the number of IAEs and PCs [26,27,28,29]. Typically, the duration of laparoscopic cholecystectomies oscillates between 80 and 100 min (BC 102.4 min; median = 96 min [75; 130]). It depends on the intraoperative conditions and the experience of the lead operator. Thus, 17.5 min make up 17–22% of the total duration of the surgery, and, clinically, these numbers make a relevant difference. A prolonged time of operation influences the increase in the risk of possible further complications and has a negative impact on operating rooms’ daily use. Our study reveals that the surgeries performed after the pandemic lasted significantly longer than the same surgeries before the break in surgical practice caused by COVID-19, with *p* = 0.024. This objective and statistically significant assessment of surgical skills may serve as evidence of surgical skill decay phenomena.

The number of IAEs did not show a significant difference between the groups in the univariate analyses. However, the greater number of acute cholecystectomies in the pre-pandemic group indicates that these surgeries were performed under more challenging conditions. Consequently, the percentage of so-called DGB, described by surgeons operating on patients before the pandemic, was noticeably higher (Table 1). This indicates that, to objectively compare the lead surgeons’ operating skills in the BC vs. AC groups, we should exclude this concomitant variable in the multivariate analysis. After the exclusion of the DGB factor in both groups, we found that the risk of IAEs substantially increased among the surgeries performed after the pandemic (AC vs. BC from aOR = 1.38 to aOR = 1.8; *p* = 0.100), nearing statistical significance. This result suggests that the pandemic period had an impact on the impairment of surgeons’ agility and some kind of surgical intuition. It appears that, to observe statistically significant differences, an analysis on a larger study group would be required. 

It has been proven that surgical skills tend to deteriorate during prolonged periods without practice [13]. Over time, even a short break in training and daily contact with surgical patients can make surgeons feel that their technical abilities have been impaired [11]. The lack of continuous practice and hands-on training leads to a decrease in self-confidence and self-assessment of clinical abilities, fostering a sense of incompetence, which can result in the actual loss of acquired surgical skills [11,13,17,20]. Our study appears to confirm the occurrence of this phenomenon not only among residents but also among specialists. We identified factors such as the speed of surgery and the number of IAEs, enabling us to objectively evaluate the magnitude of surgical skill decay phenomena.

An interesting finding is that, in group AC, the difference in the duration of surgery between the specialists and the residents did not reach statistical significance (*p* = 0.22) in contrast to group BC (*p* = 0.033). Our findings show that, due to break in normal surgical practice, the specialists after COVID-19 stepped back, close to the residents’ speed level from the period before COVID-19 (mean duration of surgery performed by specialists in the AC group 113.1 ± 39.0 vs. mean duration of surgery performed by residents BC 114.4 ± 41.0). However, the treatment effect of these patients and the number of severe postsurgical complications were not significantly different between the study groups. The prolonged surgery time may possibly be a consequence of surgeons being more cautious and having less confidence in their own skills due to much less practice during the COVID-19 pandemic period. Our findings align with the description of skill decay in a study by Bodilly et al. conducted in 1986 on military recruits, indicating that the ability to perform motor tasks in a certain time tends to deteriorate faster than the accuracy of their performance [14].

Despite the prolonged duration of surgery observed in our research among both young and experienced surgeons after COVID-19, it is noteworthy that specialists consistently performed surgeries at a significantly faster pace than residents in both study groups (BC specialists: 96.2 min ± 39 min vs. BC residents: 114.4 min ± 41.8 min; AC specialists: 113.1 min ± 39 min vs. AC residents 129 min ± 47 min). These findings substantiate our thesis that the duration of surgery can serve as an objective determinant of surgical skills. Similar conclusions were found by British scientists who established the validity of time as an indicator of laparoscopic surgery performance by demonstrating that residents had longer total operative and hepatocystic triangle dissection times compared to specialists [30].

An American paper examining the impact of the pandemic on the specialty training of surgical and anesthesiology residents from two major academic centers in New York City revealed that residents during COVID-19 failed to complete the minimum number of required surgeries, marking the first of such instances in the hospitals’ 20-year history [11]. Both residents (63%) and the specialists who train them (75%) reported a noticeable loss of surgical skills in the short period after the pandemic [11,31]. The same medical centers conducted a subjective questionnaire study on the long-term effects of COVID-19 on residents’ clinical skills. The survey showed that doctors regained their lost abilities after returning to normal surgical practice [31]. These encouraging findings highlight the transient nature of surgical skill decay as practice returns. Thus, it is necessary to assess these results through an objective study, comparing the period several months after the end of the COVID-19 pandemic, with the return of surgical departments to normal activity, to the pre-pandemic period. We would like to evaluate this in the future using parameters that our study proved to be objective, measurable determinants of surgical skills.

COVID-19 underscored the significance of external factors in shaping the quality of surgical training and the abilities that residents strive to achieve. Beyond the eagerness of trainees to improve and acquire knowledge, external factors play a pivotal role. Hence, it becomes crucial to complement surgical training with courses and coaching workshops to improve skills in performing independent surgeries [32,33,34]. Residents, after practicing procedures on simulators, are likely to feel more confident when operating on real patients, without jeopardizing their safety [35,36]. Although this approach has some limitations and cannot be a substitute for clinical practice, it serves as a valuable addition to traditional training methods.

We wish to emphasize that, with the onset of globalization, people have become more vulnerable to infections and can act as vectors, facilitating the rapid global transmission of diseases [37]. Considering this, there is a possibility that, in the following years, we may face the challenge of another pandemic. In anticipation of such an event, it is crucial to be prepared to prevent the development of surgical skill decay.

The authors acknowledge the constraints of this study, attributed to its exclusive reliance on a single-center approach and the examination of a specific surgical procedure carried out by a distinct group of surgeons. To fully assess the magnitude of the post-pandemic surgical skill decay phenomenon, multicenter research involving different cohorts should be conducted.

## 5. Conclusions

The COVID19 period did not lead to a decrease in the safety of surgical procedures. However, by defining measurable determinants of surgical skills, our study objectively shows that the reduction in the number of surgeries resulted in the occurrence of a phenomenon known as surgical skill decay.

Furthermore, the significant increase in the duration of surgery appears to be a consequence of greater vigilance among the operating teams, likely related to impaired or decreased confidence in managing and assessing intraoperative situations. Therefore, we should be aware of how important it is for a surgeon’s proficiency to continuously practice and improve skills, also by taking advantage of opportunities provided by technological advances.

## Figures and Tables

**Table 1:** Demographics and operative conditions before and after the COVID-19 pandemic.

	Before COVID-19	After COVID-19	
	(n = 76)	(n = 72)	
Demographics:	n (%) or	n (%) or	*p*-value
	median [IQR]	median [IQR]	
Sex: Male	32 (42.1)	28 (38.9)	0.69
Age (years)	51 [19–84]	54 [25–87]	0.69
Body mass (kg)	78.5 [49.5–129]	79 [50–144]	0.51
BMI (kg/m^2^)	27.2 [18.8–44.6]	28.5 [17.5–42.1]	0.36
Operative conditions:			
Acute gallbladder	27 (35.5)	15 (20.8)	**0.047**
Difficult gallbladder ^#^	53 (69.7)	34 (47.2)	**0.005**
Specialist as lead surgeon	50 (65.8)	42 (58.3)	0.35

^#^ One of the following: enlarged gallbladder, hard or thick gallbladder wall, inflammation seen during surgery, and intraperitoneal adhesions.

**Table 2:** Incidence of outcomes.

	Before COVID-19	After COVID-19	
	n (%) or Mean ± SD	n (%) or Mean ± SD	*p*-Value
Number of intraoperative adverse events:	33 (43.4)	37 (51.4)	0.33
· Injury of the gallbladder wall	15 (19.7)	21 (29.2)	0.18
· Evacuation of gallstones	1 (1.3)	5 (6.9)	0.08
· Intraoperative bleeding	21 (27.6)	23 (31.9)	0.57
Postoperative complications ^#^	10 (13.2)	7 (9.7)	0.51
Conversion	14 (18.4)	6 (8.3)	0.07
Readmission	1 (1.3)	2 (2.8)	0.53
Duration of surgery (min)	102.4 ± 40.4	119.9 ± 42.6	**0.024**
Hospitalization time (days)	6.2 ± 2.8	5.6 ± 3.3	**0.011**

^#^ One of the following: postoperative fluid collection, biliary leakage, postoperative bleeding, wound infection, and necessity of reoperation.

**Table 3:** Risk of occurrence of outcomes of interest depending on the operative conditions and characteristics of the patients both before and after the COVID-19 pandemic.

Factors of Interest	OR	95% CI	*p*-Value	OR	95% CI	*p*-Value	
	**Intraoperative adverse events before COVID-19**			**Intraoperative adverse events after COVID-19**			

Operative conditions:							
Specialist as the lead surgeon	0.85	0.33–2.19	0.73	0.70	0.27–1.78	0.45	
Difficult gallbladder	2.94	1.00–8.62	**0.049**	2.81	1.08–7.33	**0.035**	
Acute gallbladder	1.07	0.41–2.75	0.894	2.22	0.67–7.32	0.19	
Demographics:							
Sex: Male	1.28	0.51–3.19	0.61	3.97	1.43–11.0	**0.008**	
Age [per 1 years]	1.02	0.99–1.05	0.19	1.02	0.98–1.05	0.33	
Body mass [per 1 kg]	1.04	1.01–1.07	**0.016**	1.01	0.99–1.04	0.37	
BMI [per 1 kg/m^2^]	1.18	1.05–1.31	**0.004**	1.03	0.94–1.13	0.52	
	**Postoperative complications before COVID-19**			**Postoperative complications** **after COVID-19**			
Operative conditions:							
Specialist as the lead surgeon	0.47	0.12–1.79	0.27	0.50	0.10–2.42		0.39
Difficult gallbladder	4.50	0.54–37.8	0.17	1.56	0.32–7.51		0.58
Acute gallbladder	3.21	0.82–12.6	0.09	1.60	0.28–9.20		0.60
Demographics:							
Sex: Male	0.30	0.06–1.52	0.15	2.28	0.47–11.1		0.31
Age [per 1 years]	1.00	0.96–1.04	0.82	1.02	0.96–1.08		0.48
Body mass [per 1 kg]	1.02	0.98–1.06	0.29	1.00	0.96–1.04		0.93
BMI [per 1 kg/m^2^]	1.10	0.98–1.24	0.11	1.00	0.87–1.16		0.97
	**Conversion** **before COVID-19**			**Conversion** **after COVID-19**			

Operative conditions:							
Specialist as the lead surgeon	0.92	0.27–3.10	0.90	1.47	0.25–8.62		0.67
Difficult gallbladder	7.15	0.88–58.4	0.07	#	#		0.93
Acute gallbladder	10.5	2.61–42.7	**0.001**	4.50	0.81–25.1		0.09
Demographics:							
Sex: Male	4.55	1.28–16.2	**0.020**	9.35	1.03–84.9		**0.047**
Age [per 1 years]	1.01	0.97–1.05	0.59	1.05	0.98–1.12		0.18
Body mass [per 1 kg]	1.02	0.98–1.05	0.36	0.97	0.91–1.04		0.35
BMI [per 1 kg/m^2^]	1.02	0.92–1.14	0.70	0.88	0.71–1.10		0.25

# Due to the small number of patients, the estimation of risk is impossible.

**Table 4:** The risks of intraoperative adverse events, postoperative complications, and conversion on a series of two-factor analyses depending on the moment of surgery (after vs. before the COVID-19 pandemic) and one of the factors of interest.

	aOR	95% CI	*p*-Value	Factors of Interest	aOR	95% CI	*p*-Value
**Risk of intraoperative adverse events**							
After vs. Before	1.38	0.72–2.63	0.33	#			
After vs. Before	1.80	0.89–3.61	0.10	Difficult gallbladder	2.87	1.40–5.86	**0.004**
After vs. Before	1.35	0.71–2.59	0.36	Specialist as the lead surgeon	0.77	0.39–1.50	0.43
After vs. Before	1.46	0.75–2.81	0.27	Acute gallbladder	1.43	0.69–2.96	0.34
After vs. Before	1.43	0.74–2.77	0.29	Sex: Male	2.16	1.10–4.22	**0.030**
After vs. Before	1.37	0.71–2.62	0.35	Age (per 1 year)	1.02	1.00–1.04	0.11
After vs. Before	1.27	0.63–2.53	0.50	Body mass [per 1 kg]	1.02	1.00–1.04	**0.020**
After vs. Before	1.27	0.63–2.56	0.51	BMI [per 1 kg/m^2^]	1.10	1.02–1.17	**0.009**
**Risk of postoperative complications**							
After vs. Before	0.71	0.26–1.98	0.51	#			
After vs. Before	0.85	0.30–2.43	0.76	Difficult gallbladder	2.41	0.73–7.98	0.15
After vs. Before	0.67	0.24–1.88	0.45	Specialist as the lead surgeon	0.48	0.17–1.34	0.16
After vs. Before	0.82	0.29–2.34	0.71	Acute gallbladder	2.45	0.86–6.97	0.09
After vs. Before	0.71	0.25–1.97	0.50	Sex: Male	0.77	0.27–2.21	0.63
After vs. Before	0.71	0.25–1.98	0.51	Age (per 1 year)	1.00	0.97–1.04	0.81
After vs. Before	0.83	0.29–2.33	0.72	Body mass [per 1 kg]	1.01	0.98–1.04	0.49
After vs. Before	0.82	0.29–2.31	0.70	BMI [per 1 kg/m^2^]	1.06	0.97–1.16	0.21
**Risk of conversion**							
After vs. Before	0.40	0.15–1.11	0.08	#			
After vs. Before	0.57	0.20–1.64	0.29	Difficult gallbladder	14.9	1.92–116.1	**0.010**
After vs. Before	0.41	0.15–1.12	0.08	Specialist as the lead surgeon	1.08	0.40–2.93	0.88
After vs. Before	0.52	0.18–1.55	0.24	Acute gallbladder	7.64	2.66–21.9	**0.0002**
After vs. Before	0.40	0.14–1.14	0.09	Sex: Male	5.58	1.88–16.5	**0.002**
After vs. Before	0.40	0.14–1.10	0.08	Age (per 1 year)	1.02	0.99–1.05	0.25
After vs. Before	0.31	0.10–0.99	**0.049**	Body mass [per 1 kg]	1.00	0.98–1.03	0.83
After vs. Before	0.31	0.10–1.01	0.053	BMI [per 1 kg/m^2^]	0.99	0.89–1.09	0.78

^#^ Univariate analysis.

**Table 5:** Duration of surgery depending on the factors of interest both before and after the COVID-19 pandemic.

Factors of Interest	Before COVID-19 (BC)			After COVID-19 (AC)		Difference in Time (After vs. Before)
Lead surgeon:		Mean time (min)	*p*-value	Mean time (min)	*p*-value	
	Specialist	96.2 ± 39.0		113.1 ± 39.0		16.9
			**0.033**		0.22	
	Resident	114.4 ± 41.0		129.0 ± 47.0		14.6
Intraoperative conditions:						
	Difficult ^#^	111.4 ± 41.1		130.9 ± 47.1		19.5
			**0.005**		0.14	
	Normal ^#^	81.6 ± 31.3		111.2 ± 37.8		29.6
Type of surgery:						
	Acute	114.3 ± 33.9		139.2 ± 55.3		24.9
			**0.017**		0.19	
	Elective	95.8 ± 42.8		115.2 ± 38.6		19.4

^#^ Gallbladder.

## Data Availability

Data are available from the corresponding author upon request.
